# Social pharmaceutical innovation and alternative forms of research, development and deployment for drugs for rare diseases

**DOI:** 10.1186/s13023-022-02476-6

**Published:** 2022-09-05

**Authors:** Conor M. W. Douglas, Fernando Aith, Wouter Boon, Marina de Neiva Borba, Liliana Doganova, Shir Grunebaum, Rob Hagendijk, Larry Lynd, Alexandre Mallard, Faisal Ali Mohamed, Ellen Moors, Claudio Cordovil Oliveira, Florence Paterson, Vanessa Scanga, Julino Soares, Vololona Raberharisoa, Tineke Kleinhout-Vliek

**Affiliations:** 1 Department of Science, Technology and Society, 307 Bethune College, York University, 4700 Keele Street, Toronto, ON M3J 1P3 Canada; 2 University of São Paulo Public Health School, Health Law Research Center of the University of São Paulo, Av. Dr. Arnaldo, 715, São Paulo, Brazil; 3 Copernicus Institute of Sustainable Development, Universiteit Utrecht, Princetonlaan 8a, 3584 CB Utrecht, The Netherlands; 4 São Camilo Medical School, School of Public Health, University of São Paulo, Av. Dr. Arnaldo, 715, São Paulo, Brazil; 5 Mines ParisTech, Université PSL in Paris, 60 Boulevard Saint Michel, 75272 Paris Cedex 06, France; 6 Department of Science and Technology Studies, 307 Bethune College, York University, 4700 Keele Street, Toronto, ON M3J 1P3 Canada; 7 Faculty of Social and Behavioural Sciences, International School of Social Sciences and Humanities, University of Amsterdam, Spui 2, 1012 WX Amsterdam, The Netherlands; 8 Faculty of Pharmaceutical Sciences, University of British Columbia, 2405 Wesbrook Mall, Vancouver, BC V6T 1Z3 Canada; 9 Center for Social Innovation, Université PSL in Paris, Mines ParisTech60 Boulevard Saint Michel, 75272 Paris Cedex 06, France; 10 Faculty of Health Policy and Equity, York University, 4700 Keele Street, Toronto, ON M3J 1P3 Canada; 11 Innovation and Sustainability, Copernicus Institute of Sustainable Development, Universiteit Utrecht, Princetonlaan 8a, 3584 CB Utrecht, The Netherlands; 12 Public Health at the Sergio Arouca National School of Public Health (ENSP/Fiocruz), Av. Brazil, 4365 - Manguinhos, Rio de Janeiro, Brazil; 13 Osgoode Hall Law School of York University, 4700 Keele Street, Toronto, ON M3J 1P3 Canada; 14 The Federal University of Sao Paulo (UNIFESP), School of Public Health at the University of São Paulo, Av. Dr. Arnaldo, 715, São Paulo, Brazil; 15 Geosciences, Innovation Studies, Innovation and Sustainability Institute, Universiteit Utrecht, Princetonlaan 8a, 3584 CB Utrecht, The Netherlands

**Keywords:** Social pharmaceutical innovation, Orphan drugs, Rare diseases, Therapeutic research and development, Social innovation, Policy, Patient organisations

## Abstract

Rare diseases are associated with difficulties in addressing unmet medical needs, lack of access to treatment, high prices, evidentiary mismatch, equity, etc. While challenges facing the development of drugs for rare diseases are experienced differently globally (i.e., higher vs. lower and middle income countries), many are also expressed transnationally, which suggests systemic issues. Pharmaceutical innovation is highly regulated and institutionalized, leading to firmly established innovation pathways. While deviating from these innovation pathways is difficult, we take the position that doing so is of critical importance. The reason is that the current model of pharmaceutical innovation alone will not deliver the quantity of products needed to address the unmet needs faced by rare disease patients, nor at a price point that is sustainable for healthcare systems. In light of the problems in rare diseases, we hold that re-thinking innovation is crucial and more room should be provided for alternative innovation pathways. We already observe a significant number and variety of new types of initiatives in the rare diseases field that propose or use alternative pharmaceutical innovation pathways which have in common that they involve a diverse set of societal stakeholders, explicitly address a higher societal goal, or both. Our position is that principles of social innovation can be drawn on in the framing and articulation of such alternative pathways, which we term here *social pharmaceutical innovation* (SPIN), and that it should be given more room for development. As an interdisciplinary research team in the social sciences, public health and law, the cases of SPIN we investigate are spread transnationally, and include higher income as well as middle income countries. We do this to develop a better understanding of the social pharmaceutical innovation field’s breadth and to advance changes ranging from the bedside to system levels. We seek collaborations with those working in such projects (e.g., patients and patient organisations, researchers in rare diseases, industry, and policy makers). We aim to add comparative and evaluative value to social pharmaceutical innovation, and we seek to ignite further interest in these initiatives, thereby actively contributing to them as a part of our work.

## Background

The rare disease field is host to a growing number of initiatives that engage in pharmaceutical innovation in various and distinct ways. The initiatives include novel types of research and development collaborations (e.g., public–private partnerships), decentralised forms of manufacturing, alternative regulatory and reimbursement schemes, etc. They are of significance due to their role in addressing some of the well-documented challenges of availability, accessibility, affordability, and acceptability of treatments for rare diseases. For instance, the identification of prospective biochemical or even genetic targets and eventual development of compounds for these targets is inefficient in most drug innovation processes, let alone in rare diseases [1, 2].

Innovation for rare diseases is also associated with unique challenges. Historically, one of the reasons for the lack of innovation lies in small patient populations making investment less attractive to companies, and scientists. While market rationale for the development of niche products may be shifting [3], intellectual property rights and secrecy are thwarting information sharing, collaboration, and thereby research and innovation [4], and “national patent protection alone has not born out to stimulate domestic innovation” [5]. Another challenge in the rare disease space are the high prices associated with these products, with many of the most expensive drugs in the world targeting rare diseases [6]. There are numerous reasons extolled for the high cost of these drugs (e.g., the small market size, the high cost of research and development, high failure rates, industry’s need to recoup high-risk investments, amongst others). Fundamentally, however, this is an industry, and pharmaceutical companies are obligated to their shareholders to increase profits. Finally, there is a stark evidentiary mismatch between industry submissions and the existing regulatory and Health Technology Assessment structures. This mismatch causes delayed access to medicines and sometimes to poor coverage decisions.

While this is not an exhaustive list of the structural problems facing the pharmaceutical industry and its regulatory environment, it is evident that there are -at the very least- constraints on the current system to deliver on the needs of rare disease patients. Importantly, while some challenges are experienced differently globally (i.e., higher vs. lower and middle income countries), many are also being expressed transnationally, which thereby suggests systemic issues. Therefore, we pose that the current model of pharmaceutical innovation alone will not deliver the quantity of products needed to address the unmet needs faced by rare disease patients, nor at a price point that is sustainable for healthcare systems.

In the face of these challenges, we are observing a significant number of innovative initiatives in pharmaceutical innovation that depart from -and sometimes disrupt- the entrenched, traditional, linear, industry-led model of innovation [7]. In many jurisdictions, rare disease patients, patient organisations, and patient advocacy groups play an increasing role in all phases of drug research and licensing [8, 9]. Transformations are also underway in drug manufacturing and production processes, with pharmacists increasingly compounding medications and in-hospital production of drugs for individuals or groups of patients with rare disease [10–12]. At the regulatory level, an increasing number of outcomes-based risk-sharing agreements have emerged to help manage the uncertainty of drug efficacy through further collection of data. Similarly, finance-based risk-sharing agreements have worked to manage the impact of drugs for rare diseases on the sustainability of health systems through price controls. Risk-sharing agreements have also become more prevalent after market approval, in the form of extended pharmacovigilance procedures and the collection of real-world evidence to help inform coverage decisions [13–19].

These initiatives in pharmaceutical innovation just mentioned have in common that they involve several societal stakeholders, explicitly address a higher societal goal, or both. Examples include M4K Pharma, a Canadian-based organisation forwarding a new business model “that aims to align diffuse academic and industry research into a collaborative open science drug discovery programme” [20]. It is currently focused on research and development (R&D) of a treatment for a rare paediatric brain cancer through diffused intrinsic pontine glioma (DIPG) [21]. A second example is the international platform ‘myTomorrows’, which seeks to connect patients with unmet medical needs to expanded access programmes and ongoing clinical trials [22]. A third example from later in the life-cycle of rare disease treatments is the increasing number of Latin American countries that are making use of expedited regulatory review and/or reliance pathways that use data from other jurisdictions to expedite approvals [23].

While many of the foregrounded initiatives of pharmaceutical innovation involve a wide range of stakeholders, they are often unconnected from one another and exist in a fragmented landscape. Moreover, significant variation exists in terms of organisation, and the societal goals of these initiatives range from addressing specific development barriers for particular patient populations to striving for broader systemic change. As a result, we pose that there is an urgent need for re-thinking innovation and that more room should be provided for new innovation pathways. Our position is that principles of social innovation can be drawn on in the framing and articulation of such alternative pathways, which we term here *social pharmaceutical innovation* (SPIN), and that it should be given room to be experimented with. We will use our background in the social sciences, law, and public health to develop an interdisciplinary approach to analyse -and where possible support- these initiatives in collaboration with the people and organisations involved.

To be sure, SPIN initiatives are themselves rare, that is: exceptions to the existing bio-pharma-led model for innovation dominating the landscape. That said, we see room for more alternative approaches within that innovation landscape, and interesting initiatives are underway that are doing things differently. The research program that we are advancing in the below suggests that we can learn from -and support- those initiatives through systematic interdisciplinary social science research grounded in social innovation. Our objective here is to outline that research program while setting the stage for future empirically-derived policy recommendations to emerge from on-going research.

## Towards social pharmaceutical innovation

### Pharmaceutical R&D in rare diseases as an interactive and multi-faceted process

Over the years, studies of science, technology and society (STS), law and the emerging field of innovation studies have documented the inseparability of social and technical aspects of the world and how they co-develop. Extensive research across a wide range of fields suggest that technological change is best understood when we analyse it as evolving in multi-directional and iterative forms rather than a linear manner. As such, the evolution of new technologies shows the essential -yet variable- role of involved social groups in shaping innovations [24–26]. Even in the context of pharmaceuticals, where process stages are tightly structured and heavily regulated, there is not one predetermined channel or finite manner through which drugs are researched, developed, and brought to market. On the contrary, in the area of pharmaceuticals numerous routes to innovation can and do exist [27–31]. Understanding the variety and form of these pathways requires an understanding of the social and technological factors that shape them.

Quite clearly then, science and technology do not exist in a social, historical, cultural, political, or economic vacuum. Institutional arrangements, laws, policies, economic and ethical assessments vary from one country to the next and impact how and which biotechnologies are developed [32]. The perspective of innovation processes being non-linear and interactive works to demonstrate how these factors influence emerging forms of pharmaceutical innovation in the rare disease space. At the same time, innovations in health technologies and medical science also impact the manner in which our societies are organised, how we relate to each other, and how we see ourselves. Examples here include ending diagnostic odysseys through advanced genomic technologies that can have both positive and negative effects for rare disease patients and their families by transforming an undiagnosed child into a rare disease patient, thereby perhaps ending hope of recovery while also facilitating connection to peer groups and community building [33]. Another example is how variable access to drugs for rare diseases can create or exacerbate (social) inequalities between rare disease patients, as well as divisions with patients receiving treatment for more common conditions [34, 35].

Seen this way, science, technology, society, and social change should be analysed as being co-produced [36], and that obviously includes the changing field of rare diseases where we have undertaken considerable research [25, 37–48]. Our interdisciplinary approach to co-production is rooted in academic disciplines of STS, law, public health, and innovation studies. We hold that a focus on co-production is critical to develop a broader, more comprehensive understanding of how novel initiatives are seeking to address some of the challenges associated with the development of drugs for rare diseases and identifying why some of them succeed whereas others struggle.

### From social innovation to social pharmaceutical innovation

With the view of innovation being co-produced, one particularly fruitful concept for developing an understanding of these novel initiatives in rare disease research is *social innovation*. Social innovation is especially used in the context of so-called ‘wicked problems’, such as climate change, increasing life expectancy and associated health and social care costs, and growing inequalities. These problems are all characterized by complexity, interconnectedness, and “multiple and contradictory analyses and diagnoses” [51], which are certainly recognisable in the rare disease field. Inability to address these problems is accompanied by “a collapse in trust in the status quo—as established models and social relations have increasingly failed to deliver well-being for many” [51].

Social innovation can be defined as “the development and implementation of new ideas (products, services and models) to meet social needs and create new social relationships and collaborations” [49]. Westley and Antadze expanded upon this by noting that:

“Social innovation is a complex process of introducing new products, processes or programs that profoundly change the basic routines, resource and authority flows, or beliefs of the social system in which the innovation occurs. Such successful social innovations have durability and broad impact” [50].

At its core, social innovation (SI) “is aimed at improving human well-being” [49]. It is orientated towards serving social needs and towards building resilience. It is both innovation in, and innovation through, new arrangements and ways of organising. Thus, SI is concerned with both actions and their effects, and the way in which an outcome is achieved matters in that “innovation is both a process and a product” [56]. It is important to note that SI is not new; rather, there is a long history of processes and practises operating under different labels that can be traced back to the eighteenth [52] or nineteenth century [53]. Some SI scholars have argued that it is, in fact, “a common dynamic of human history” [54].

While (whole) systems thinking is of use and value for understanding the dynamics related to complex and interconnected phenomena like the environment [55]. SI is often studied on a project or organisational level, which aligns well with our focus on initiatives in the rare disease field. At the same time, SI projects are heavily linked with various parts of (innovation) systems [51]. SI is generally needs-led or demand-led rather than supply-driven, which translates to significant roles for users and citizens in innovation processes [49]. For this reason, SI can be characterised more as “grass roots”, “bottom-up” and community-supported compared to more conventional forms of innovation [56]. Innovation that is bottom-up in nature, with a significant role played by users, flourishes within open and collaborative approaches [56]. Openness, in this context, refers to the more freely sharing of knowledge, a more communal approach to the ownership of knowledge, as well as disciplinary openness in which different approaches can be integrated together towards problem solving [49]. It is often seen as critical that diverse actors from a broad range of stakeholder groups or sectors are involved in exchanging ideas and values towards the generation of solutions [57].

When diverse stakeholders are brought together in open and collaborative problem-solving initiatives, much stands to be gained in terms of the products or outcomes of these collaborations [58]. In doing so, SI is as much directed at capacity building and empowerment of users and citizens [57] as it is to tailor-made results to specific needs instead of mass-produced solutions to more general problems [49]. Importantly, products resulting from SI are not solely market-driven; to the contrary, “social innovations [often] literally serve demands which neither the state nor markets would or can meet” [56]. This is not to say that SI does not involve businesses or private capital; rather, “new business models [are emerging] that meet the needs of underserved populations” [58]. Here capital investments are not exclusively focussed on maximising their returns, and businesses can be involved in collaborations. To this end SI is socially orientated, it is directed at developing resilience among institutions, networks, and systems, as well as “enhance[ing] an individual’s capacity to act” [49] based on values of solidarity and inclusiveness.

Well-known examples of social innovation are technologies like M-PESA, which is a form of mobile banking used in low- and middle-income countries allowing users to easily save and transfer money in the absence of conventional bank accounts. Scholars consider M-PESA a social innovation on the institutional level as it reconfigures market structures and patterns [51]. In fair trade, another well-known social innovation, marginalised farmers are connected to ethically-minded consumers through novel product distribution processes that seek to reduce global inequality and deliver additional social value [59]. Prominent examples of health-related social innovation projects include the deployment of community-driven diagnostic techniques for malaria testing via schools in Malawi, cervical human papillomavirus (HPV) sample self-collection in Peru, and crowdsourcing human immunodeficiency virus (HIV) testing in China [60], and the world's largest provider of cataract surgery in India (i.e., Aravind Eye Care) that provides “low-cost products and services to the poor…[by combining] a hyper-specialised division of low- and high-skilled labour that is unheard-of in costly hospitals of the industrialised world” [61]. SI may also have indirect health effects, such as projects contributing to Sustainable Development Goals that seek to improve overall health and well-being [62–64].

#### Defining social pharmaceutical innovation (SPIN)

The novel practices we are observing across the R&D life-cycle of rare disease drugs are creating opportunities for re-envisioning pharmaceutical innovation through what some SI scholars refer to as the “adjacent possible” [54, 65, 66]. This term refers to “the range of alternative social arrangements [which are] just beyond the horizon of prevailing practice” [54]. Social pharmaceutical innovation (SPIN) can be regarded as a way to both more fully understand these “adjacent possibles” in drug development, as well as contribute to its further progress.

To explore SPIN, we need a working definition. We understand SPIN as novel forms of collaborative processes, programs, policies, procedures and/or designs involving diverse sets of actors that break with conventional pharmaceutical innovation practices for the production of safe, effective, and accessible interventions that address unmet societal needs of rare disease patients and that are not primarily market driven. Similar to SI, we see SPIN pertaining to both transformations in processes as well as in novel outcomes; however, these two concepts differ in the respect that we see SPIN as an emerging techno-social phenomenon and a research object rather than an analytical perspective. We see SPIN as a ‘working concept’, both in terms of the work it carries out as a heuristic device that aids in framing research and asking pertinent questions concerning transformations in pharmaceutical R&D, and in terms of being a concept ‘in work’ in terms of its evolving nature. As such, we anticipate our definition of SPIN to develop through further empirical investigations, conceptual elaboration and engagement with stakeholders.

By framing novel initiatives in rare diseases in terms of SPIN, we aim at developing a better understanding of the field’s breadth as well as exploring contributions to—and further opportunities for—change ranging from the clinical to system levels. Furthermore, we seek to identify commonalities between initiatives in a fragmented landscape and this lens can be instructive in making sense of the organisational processes and goals of rare disease initiatives. In doing so, a SPIN framing also contributes to creating/identifying a common language to understand phenomena and enables communication about them, thereby making innovative processes and products more visible, legible, and comprehensible.

### Social pharmaceutical innovation: a tentative typology

To build-up this project and to facilitate its further collaborative development, we outline three types of SPIN that our case studies deal with, which brings a range of important questions into focus. These different types of SPIN reflect diverse points throughout the life-cycle of pharmaceutical research, development and deployment, which allows us to examine innovation challenges in rare diseases in terms of whole systems. Furthermore, these types of SPINs that we outline represent initiatives that are tentative solutions to some of the challenges facing rare diseases.

The first type of SPIN to consider are *novel R&D partnerships across the public, not-for-profit and private sectors*. These forms of SPIN exemplify the critical role that collaboration stands to play in rare disease research in terms of creating networks, connections, and cooperation, which includes the importance of patient empowerment in developing and steering research based on their needs. These partnerships can, and do, cover the full range of research from the very upstream developments of novel technological platforms, systems, and policies for the sharing of genomic data for gene discovery to further diagnostics (e.g., Canadian Genomics4rd research platform [67] or European Share4Rare platform [68]), partnerships that focus on N-of-1 trials (i.e., trials on a very small number of patients, and even on a single patient) [69] and the development of new drugs (e.g., Inspire2Live, a Dutch cancer patient organisation in the process of co-creating a clinical trial [70]), to the repurposing of existing drugs [71]. Other partnerships have developed around clinical research that combines clinical data and observations reported by patients and/or their representative organisations when the clinical outcomes are difficult to assess due to the rarity of the disease. Examples of this can be seen through the French Muscular Dystrophy Association (AFM) who argue that classic endpoints do not capture all benefits that treatments bring to patients, which are not listed as an endpoint despite being very valuable for patients (e.g., some treatments for neuromuscular diseases may help patients move one finger and manipulate the controls of their electric wheelchair). Central questions raised in understanding this first type of SPIN concern the nature of multi-sectoral partnerships in question, in terms of what they do and what they aim for. Also critical to an appreciation of this form of SPIN is describing how the various actors involved frame the problems and causes they seek to address (e.g., right to health, social justice, equity, unduly high profits for companies), and how they reflect on their role in what they are doing, why they are doing it, and how their practices align with their initial motivations and incentives. Understanding these partnerships means understanding how they organise work and activities relative to the medical, practical, regulatory, and politico-economic environment, and the obstacles they face as they proceed.

A second type of SPIN we have started to study is the development of *alternative forms of provision and licensing*. These include magisterial preparations (i.e., medicines prepared by pharmacists based on prescriptions for unmet needs like lower dosages for children, but also when negotiations for lowering a drug’s price fail), public sector manufacturing (e.g., when the state or a public–private partnership takes the lead in producing a treatment in their own facilities) (72), early access schemes [73] and compassionate use (e.g. the provision of promising experimental treatments before they get market approval in the context of urgent medical needs), and adaptive pathways (e.g. the European Medicines Agency’s approval under exceptional circumstances and conditional marketing authorisation). In exploring these alternative forms of provision and licensing, the nature of scientific and economic evidence produced throughout SPIN is brought forward, as well as the sort of knowledge this evidence is based upon. Critical here is understanding how evidence is debated between the various actors involved, in particular when evidence is brought in by patient organisations, and the extent to which this evidence challenges the statistical reasoning that underlies clinical trials and much Health Technology Assessment. Another question is whether and how actors from different institutional backgrounds (e.g., public, private, community) are able to align their incentives and activities in novel collaborative arrangements?

The third type of SPINs studied are *alternative regulatory frameworks for coverage*. Initiatives for new medico-economic Health Technology Assessment procedures that consider the social value of drugs for unmet needs [74, 75], as well as new pricing [76] and reimbursement schemes negotiated between companies and public authorities to lower the prices of certain drugs, are some examples [19, 77, 78]. Here the focus is on the nature of regulatory and institutional change that SPIN contemplates, or drives. In particular, interest centres on how these alternative regulatory frameworks for coverage disrupt the traditional linear model of pharmaceutical innovation, and in some instances conjointly address issues of availability (e.g., R&D and clinical trials), accessibility (e.g., pricing and coverage), and acceptability (e.g., safety vs. mortality of disease and equity issues). It is important to highlight the role of modern democracies to create legal and institutional responses to guarantee innovation and accessibility of pharmaceuticals for rare diseases.

While not an exhaustive typology, these diverse forms of SPIN demonstrate that innovations are needed -and are underway- throughout the research and development life-cycle. Tackling the challenges facing pharmaceutical R&D for rare diseases requires a whole system lens to identify dynamics related to early stage research and development, production, and manufacturing, as well as the coverage and payment issues raised by questions of “value” that override downstream issues of pricing and coverage [79]. Quite clearly, the nature of partnerships in early-stage research and clinical trials impinges on how products stand to be manufactured, brought to market and paid for.

Furthermore, it is also quite clear that the answers to the questions associated with the three different types of SPIN outlined above vary across the countries of the cases explored by the respective research teams on which our project builds (i.e., Brazil, Canada, France and the Netherlands). Discussion of similarities and differences between cases -as well as national contexts- on the basis of three cross-cutting issues helps to further articulate the integrated framework our work seeks to promote. It is a discussion of those cross-cutting issues that we now turn to in the development of our analytical framework for SPIN.

### Towards an analytical framework for social pharmaceutical innovation

Explorations into different types of SPIN necessitate analysis on at least three cross-cutting issues. The first is an understanding of the diverse problem-framings and goals of SPIN initiatives. This includes the national and transnational debates surrounding drugs for rare diseases, as well as how these debates are framed, and through which media they play out. In part, this entails understanding how various actors frame the problems and the causes that their innovative efforts seek to address, and what they attempt to accomplish through new forms of collaboration. What issues do particular groups bring into the spotlight, and how do they seek to address them through their SPIN initiative? Is it possible to identify areas of convergence or divergence of problem-framings and/or goals within these novel forms of collaborative research? Are actors working towards addressing the same issue or is the collaboration a marriage of convenience? In forms of SPIN that involve alternative forms of provision and licensing, analytical questions include how intellectual property (IP) regimes are being framed. Our approach seeks to understand how a SPIN would approach IP: as a driver for innovation, or as a constraint that is locking-in particular modes of manufacturing and delivery? Furthermore, interest here centres on how SPIN projects are approaching issues of local capacity, both in terms of the capital, technology, and facilities required for the production of advanced products that target rare diseases (e.g., cell and virus manufacturing) as we as human capital, personnel, and advanced training needed to actually carry out the manufacturing work. An appreciation of these problem-framings is not only instructive for understanding the goals of SPIN initiatives, but also to understand if and how much room there is for differential IP regimes and alternative manufacturing capacities. For SPINs targeting alternative regulatory frameworks for coverage, understanding how participants see rare disease policy initiatives across different constituencies is also critical for identifying and characterising the target of their policy interventions. Some alternative frameworks for coverage have recently been attempted to advance equity in terms of access across European member-states as well as between Canadian provinces. Other innovative initiatives target the cost containment on drugs for rare diseases and to ensure the sustainability of health systems more broadly. Alongside the characterisation of these policy goals, we also investigate how bureaucratic and political frameworks constrain novel forms of R&D and medicinal products. Could alternative regulatory frameworks for coverage disrupt the traditional linear models of pharmaceutical innovation? How? To what extent, but also why not? Answers to such questions are key to fully understanding the forms and limits of social pharmaceutical innovation and to properly articulate policy recommendations for (experimentation with) SPIN initiatives.

The second key cross-cutting issue is processes: how are SPIN constituted and through what processes and factors are they adjusted and actually carried out? What role does the decentralised and distributed character of these SPIN processes play in this, and how are collaborations organised and managed? Careful consideration must be given to when, where and how multi-sectoral partnerships emerge, and particularly when patient organisations intervene and/or are mobilised for the design and conduct of SPIN. Studying SPIN means attending to the processes through which patients and external publics and media are involved. It also requires analysing how nascent forms of R&D produce new forms of evidence that challenge existing regulatory structures and motivate institutional reform and change. Through such R&D new forms of SPIN may link up with the associated battles and debates over new forms of evidence. This is especially likely when evidence is brought in by patient organisations or investigator-initiated trials or registries. Here, research focuses on the extent to which this evidence challenges the statistical reasoning that underlies clinical trials regulations and Health Technology Assessments that are central to the authorization of drugs for rare diseases. Subsequent analysis along the life-cycle of pharmaceuticals then focuses on the nature of regulatory and institutional change that SPIN contemplates or drives, and in some instances, overturns. Significant variation exists across constituencies and regulatory domains with regards to patient access and health insurance coverage. Health costs coverage regimes, risk sharing agreements and alternatives to existing systems that can be understood as SPINs are topics to be covered in our research. How these agreements are negotiated and carried out is by no way uniform, which offers fertile ground for cross-national learning and comparative analysis of the socio-institutional character of SPIN processes, for which input from the rare diseases field is most welcome.

Finally, SPINs must also be examined and held to account for their outcomes and/or products. Critical questions must be asked of the extent to which SPINs are delivering on their promises: What successes may be claimed? What can be learned? How to improve the track record of SPINs in targeting rare diseases? How and where have SPINs reorganised work and activities relative to the medical, practical, regulatory and politico-economic environment? When and how can activities be aligned better, and sincere collaborations be stimulated? In setting up partnerships, what obstacles do SPINs face? In summary: Are SPIN initiatives conjointly addressing the issue of availability (R&D and clinical trials) and the issue of accessibility (pricing and coverage)? What are the transformative prospects for the rare disease field as a whole?

Through these three cross-cutting lines of inquiry we may begin to think about ways of systematically analysing, assessing and ultimately understanding the different types of SPIN as introduced in “Social pharmaceutical innovation: a tentative typology” section. We invite collaborative research with stakeholders in this area on these three cross-cutting lines of inquiry. Questions that can be posed are suggested in Table 1 below, and together with initial points of discussion addressed in the section “Initial points of discussion relating to social pharmaceutical innovation”, a SPIN research program can be developed as a way of stimulating more thought and collaborative research in this area.

**Table 1:** Analytical questions to ask about social pharmaceutical innovations

LINE OF ANALYSIS	TYPE OF SPIN		
	Novel R&D partnerships across the public, not-for-profit and private sectors	Alternative forms of provision and licensing	Alternative regulatory frameworks for coverage
Problem-framing & goals of SPIN initiative	How do various stakeholders involved frame the problems -and their causes- they seek to address?	How are IP regimes locking in particular modes of manufacturing and provision? What room is there for distinct forms of IP regimes and manufacturing capacities within the national or regional context in which SPINs are taking place?	How are regulatory and political frameworks constraining novel forms of R&D and emerging forms of medicinal products? How do alternative regulatory frameworks for coverage disrupt the traditional linear model of pharmaceutical innovation?
Nature of SPIN processes	What is the nature of multi-sectoral partnerships in question (e.g., distributed, decentralised, transdisciplinary), in terms of what they actually do and what they aim for?	How is evidence debated between the various actors involved? How do diverse forms of evidence challenge the statistical reasoning that underlies clinical trials and health technology assessment?	What is the nature of regulatory and institutional change that SPIN contemplates or drives?
Outcomes of SPIN activities	How have partnerships (re)organised work and activities relative to the medical, practical, regulatory and politico-economic environment? How lasting are the R&D partnerships facilitated by SPINs? Are they one-off cooperatives or more enduring relationships?	What are the outcomes of alternative forms of provision and licensing in terms of changes in access and availability to drugs for rare diseases?	How have alternative policy and regulatory frameworks facilitated transformations in addressing the issue of availability (i.e., R&D and clinical trials) and/or the issue of accessibility (pricing and/or coverage)?

### Initial points of discussion relating to social pharmaceutical innovation

Through the framing of examples of SPIN in the rare disease space, important differences are emerging between seemingly identical cases and across contexts and constituencies. The first type of difference relates to the diversity of initiatives. As we have argued, SPINs cannot easily be divided up into discrete sets of practices, but rather amount to a series of distinct and different initiatives and dialogues ranging from more inclusionary forms of research and development to policy changes at the regulatory or health systems level. This rich arena offers ample and significant opportunities to learn how different constituencies engage with and address challenges relating to drugs for rare diseases. This diversity prompts social scientific methodological reflection in terms of our topics of study (i.e., actions, processes or discourses). Related to this point of diversity of SPIN is the fact that writ-large differences exist depending on global, national or even regional contexts. We are observing that capacities for collaborative research and development initiatives differ between lower, middle, and higher income countries. Political stability, economic and industrial development, social inequalities, corruption, among other factors, have a direct impact on the country’s capacity for social pharmaceutical innovation. At the same time, inequalities and hurdles can also facilitate creative social innovation. Furthermore, constitutional and policy arrangements of individual countries are also proving to be significant mitigating factors in the success of—let alone possibility for- some forms of SPIN.

The second point to critically reflect on as part of what we do concerns the nature of participatory research activities in terms of who is included in such endeavours, who is left out, who decides who participates, and to what end? Here, we will focus our discussion on the role of the government/authorities and their legitimacy vis-à-vis the pharmaceutical and biotech sectors that have usually driven research and development in this area. Related to discussions about inclusion are points related to prioritisation: why are some kinds of SPIN undertaken rather than others? Why is it that advanced therapeutics such as gene and cell therapies seem to be prioritised over other types of medicine? Why do particular rare diseases draw more attention and galvanise novel research partnerships or negotiate managed access where others do not? To be sure, scientific and technical features figure into the answers to some of these questions, which our consortium considers alongside socio-cultural and political-economic drivers. How do such features and drivers relate or get related?

The third point raised in our analysis of SPIN has to do with the extent and degree of change they enable on a systemic level. Within the SI literature, it is argued that “[t]o achieve broad, lasting change, social innovations must cross multiple scales” [54]. We are indeed observing scale-up difficulties in some instances of SPIN (e.g., with hospital-based or public sector manufacturing of cell therapies). Such difficulties are the result of existing regulations, push-back from incumbent actors and/or the complexity of de- and re-contextualising solutions that work well in specific settings. However, this raises broad strategic questions for stakeholders about outcomes, success, and possible failure. How to challenge and change the system and how radical system change may be achieved? Is it the case that SPIN must have lasting system impacts if -for example- they are able to secure medicine for (a) patient(s) that might not otherwise be treated? How does one evaluate more micro-level interventions that might be transitory against endeavours that seek more system-wide change? Might we see these ‘smaller’ interventions as kernels of radical novelty (perhaps remaining mostly within the realm of exploratory scientific research) which should be cherished as potential future niches of change? Relatedly, should all niche initiatives have the ambition to diffuse or scale up? Furthermore, should more ephemeral SPIN initiatives be considered failures due to their lack of lasting change, or is there nuance in initiatives that do not scale up or even do not fully work out?

These points of discussion require careful consideration from multiple angles, which is a further reason why we actively seek collaborations with actors within these emerging initiatives in the rare disease space and input from those communities on the further development of future research agendas and aims.

## Conclusion

Pharmaceutical innovation is highly regulated and institutionalised with innovation pathways that are firmly established. As deviating from these innovation pathways is thus difficult, the currently dominant model of pharmaceutical innovation as such will not deliver the quantity and quality of products needed to address the unmet needs faced by rare disease patients, nor at a price point that is sustainable for healthcare systems. In light of the problems in rare diseases, our position is that there is a need for re-thinking innovation, that room should be provided for new innovation pathways, and that principles of social innovation can be drawn on in the framing and articulation of such alternatives. Changes are already underway around rare diseases related to pharmaceutical R&D, the organisation and delivery of health care, and increasing participatory and stakeholder-driven citizen/patient engagement. Taken together we have termed these: *social pharmaceutical innovation.* The initiatives we observe are underway, and while they may not supplant dominant modes of pharmaceutical R&D, they may offer viable alternative innovation pathways that provide novel and prospectively beneficial outcomes for the existing challenges and hurdles in this area. Building on research and practice in social innovation, our perspective on these developments seeks to add, first, explanatory value of what is taking place from a broader socio-technical perspective; second, comparative value of what has and has not worked in other contexts (including experiences with challenges and barriers within and between lower, middle, and higher income countries); and third, evaluative value concerning the outcome of these initiatives and what they have been able to achieve vis-à-vis their goals and impacts on dominant pharmaceutical R&D practises. Taking stock of these developments works not only to unite these disparate innovations, but may also provide a better and distinctive socio-technical analytical framework for understanding, explaining, and helping to improve their impacts and implications. As outlined in Table 1, by formulating these developments in terms of *social pharmaceutical innovation*, critical research questions emerge about the problem-framing and the goals through which SPINs work as well as the nature of SPIN processes and outcomes.

We see these questions and our perspective as a part of an ambitious agenda for future research that we want to contribute to and with which we hope to draw in others to do the same. We invite collaboration within this agenda, and offer our social science perspective. Furthermore, by naming and framing these activities in terms of *social pharmaceutical innovation*, we not only seek to ignite further interest in these questions but also hope to actively contribute to them through the development of shared language and concepts. We also seek to contribute by engaging with the organisations and stakeholders we study to discuss our findings. As part of this, we will organise an outreach conference on the 9th and 10th of March, 2023. There we will share the preliminary findings of our research on a selection of case studies of different types of SPINs from our respective countries, and receive feedback from stakeholders in this space on the policy recommendations we are developing in support of SPIN.

To make an impact, it is important to analyse how the differentiated dynamics of broader fields affect and structure opportunities and limitations of what can be achieved and the disagreements and struggles about what (can)not be achieved. Studying this in depth requires collaboration with actors striving for change who are involved in concrete projects as well as with experts from a variety of disciplines. We invite and welcome such collaborations, and offer our interdisciplinary expertise and perspectives there within.

## Data Availability

Not applicable. This manuscript does not contain any data.
